# Optimization and Validation of Reverse Transcription Recombinase-Aided Amplification (RT-RAA) for Sorghum Mosaic Virus Detection in Sugarcane

**DOI:** 10.3390/pathogens12081055

**Published:** 2023-08-18

**Authors:** Fenglin Wang, Qinmin Liang, Rongman Lv, Shakeel Ahmad, Mishal Bano, Guangzhen Weng, Ronghui Wen

**Affiliations:** State Key Laboratory for Conservation and Utilization of Subtropical Agro-Bioresources, College of Life Science and Technology, Ministry and Province Co-Sponsored Collaborative Innovation Center for Sugarcane and Sugar Industry, Guangxi Key Laboratory of Sugarcane Biology, Guangxi University, Nanning 530004, China; 15978182150@163.com (F.W.); m13457410861_1@163.com (Q.L.); 2208301042@st.gxu.edu.cn (R.L.); shakeel@gxu.edu.cn (S.A.); mishallbano775@gmail.com (M.B.); 18894615887@163.com (G.W.)

**Keywords:** recombinase isothermal nucleic acid amplification, RT-RAA, sugarcane, sorghum mosaic virus, sugarcane mosaic disease

## Abstract

Sorghum mosaic virus (SrMV) causes sugarcane mosaic disease and has significant adverse economic impacts on the cultivation of sugarcane. This study aimed to develop a rapid isotherm nucleic acid amplification method for detecting SrMV. Specific primers were designed to target the conserved region of the P3 gene of SrMV. The reverse transcription recombinase-aided amplification (RT-RAA) method was developed by screening primers and optimizing reaction conditions. Comparative analyses with RT-PCR demonstrated that the RT-RAA method exhibited superior specificity, sensitivity, and reliability for SrMV detection. Notably, using a standard plasmid diluted 10-fold continuously as a template, the sensitivity of RT-RAA was 100-fold higher than that of RT-PCR. Moreover, the RT-RAA reaction displayed flexibility in a temperature range of 24–49 °C, eliminating the need for expensive and complex temperature control equipment. Thus, this method could be utilized at ambient or even human body temperature. Within a short duration of 10 min at 39 °C, the target sequence of SrMV could be effectively amplified. Specificity analysis revealed no cross-reactivity between SrMV and other common sugarcane viruses detected via the RT-RAA. With its high sensitivity, rapid reaction time, and minimal equipment requirements, this method presents a promising diagnostic tool for the reliable and expedited detection of SrMV. Furthermore, it indicates broad applicability for successfully detecting other sugarcane viruses.

## 1. Introduction

Sugarcane mosaic disease (SMD) is one of the most common sugarcane virus diseases. Current sugarcane viruses that can cause SMD include sorghum mosaic virus (SrMV), sugarcane mosaic virus (SCMV), and sugarcane streak mosaic virus (SCSMV), with SrMV being the main pathogen causing SMD [1]. SrMV infects numerous gramineous plants, including sugarcane, corn, and sorghum, and is mostly spread through aphids in a non-persistent way [2]. Infected sugarcane leaves have sporadic yellow and green patches, particularly near the base of young leaves, and reduced sugarcane yield and sugar content [3]. Many studies show that SrMV exists widely in the world and can become prevalent in some sugarcane planting areas. Guangxi is the largest sugarcane-producing province in China and has instances of severe sugarcane disease, including SrMV. The detection rate of the mosaic virus in some major sugarcane growing areas in Guangxi was 75%, and the incidence rate of SrMV was 68.8% [4]. Sugarcane-producing areas in Guizhou, China, reported a SrMV detection rate of 75% [5]. The virus detection results of 104 leaf samples of *Saccharum* spp. hybrids from several provinces in China showed that the detection rate of SrMV reached 72.1% [6]. Among the new sugarcane varieties planted in the national regional trials, 77 sugarcane leaf samples with mosaic symptoms were collected, and the detection rate of SrMV was 27.3%, which implied that new sugarcane varieties in the future will still have a high risk of infection with SrMV [7]. The virus infection of chewing cane grown in Southern China was investigated, and the results showed a very serious situation. In all 106 samples collected, the detection rates of SrMV, SCMV, and SCYLV were 97.2%, 98.1%, and 95.3%, respectively, and almost all samples were co-infected with two or three different viruses [8]. The results of a recent survey show that among 901 sugarcane leaf samples with mosaic symptoms from eight provinces in China, the detection rate of SrMV was 70.1% [9]. Virus investigations in a breeding and clonal propagation trial in Louisiana, USA, showed that 70% of planted rows indicated clusters of SrMV infection, suggesting that SrMV was transmitted via cane stems [1]. A survey of the sugarcane virus disease in Tucuman, Argentina, showed that 63.2% of sugarcane leaves with mosaic symptoms were infected with SrMV, and most of them were co-infected with SCMV [10]. Although sugarcane with mosaic symptoms has a higher probability of being infected by SrMV, it is impossible to precisely identify whether a plant is infected with the virus based just on the symptoms observed in the sugarcane plant [11]. Moreover, conventional detection methods rely mainly on expensive laboratory equipment and time-consuming reaction processes. Therefore, there is a need for a rapid, field-based method for SrMV detection.

Traditional detection methods used for SrMV include real-time fluorescent quantitative RT-PCR, conventional RT-PCR, reverse transcription loop-mediated amplification (RT-LAMP), and indirect ELISA detection [12,13]. However, these methods have limitations too, and the detection cycle might take up to an hour since they require sophisticated laboratory equipment and costly temperature-controlled amplification devices. In addition, a more advanced nanobiosensor has also been established for the detection of SrMV, which can be completed in a few minutes but still requires a relevant fluorescence observation device [14].

In the RAA reaction system, the recombinase, single-strand binding protein and DNA polymerase can exponentially grow a small number of target fragments at a constant temperature [15]. First, the recombinase and primer form a complex; this complex is then located at the target sequence complementary to the primer, and the single-strand binding protein stabilizes the structure, leading to the formation of a D-LOOP structure where DNA becomes partially separated, approaching double-stranded DNA. Then, the DNA polymerase initiates the extension of a new strand from the 3’ end of the primer [15]. The RAA reaction only requires a pair of primers, simplifying the primer design process, and does not necessitate specialized thermal cycling equipment. The incubation of the reaction can be completed within 10 min under isothermal conditions at 24–49 °C. These distinctive features make RAA technology a rapid, specific, and efficient detection tool for identifying pathogenic microorganisms [16,17,18]. This method offers the potential for quick, field-based SrMV detection.

In this study, the RT-RAA detection method was used for the detection of SrMV in sugarcane. Seven primers were screened to find the best primer pair. Reaction time and temperature were adjusted based on the selected primers. The sensitivity and accuracy of this method were further evaluated by comparing it with the widely used RT-PCR technique.

## 2. Materials and Methods

### 2.1. Test Materials

The sugarcane test samples, with or without SrMV, were collected from the greenhouses of Guangxi University and the sugarcane fields in the Fusui Agricultural Science New City, Base of Guangxi University. The negative control was taken as a healthy sugarcane sample from the State Key Laboratory of Conservation and Utilization of Subtropical Agricultural Biological Resources of Guangxi University.

### 2.2. RNA Extraction and cDNA Synthesis

Total RNA was extracted from sugarcane leaf samples using the RNAiso Plus-Total RNA Extraction Reagent kit, following the provided extraction procedures. The quality and concentration of the extracted RNA were assessed using a Nanodrop ultra-micro spectrophotometer (Thermo Fisher Scientific Company, Waltham, MA, USA), with a 260/280 ratio ranging between 1.9 and 2.1. To determine the integrity of the total RNA, electrophoresis was performed on a 1% agarose gel. The extracted RNA samples were stored at −80 °C for future use.

For cDNA synthesis, the HiScript^®^ III 1st Strand cDNA Synthesis Kit (+gDNA wiper) from Nanjing Novizan Biotechnology Co., Ltd. (Nanjing, China) was utilized following the manufacturer’s instructions. The synthesized cDNA was stored at −20 °C.

### 2.3. SrMV-Positive Sample Screening

Conventional PCR primers were utilized to screen positive SrMV samples by using cDNA as a template. The PCR product obtained in this procedure was selected as the target of subsequent research.

### 2.4. RT-RAA Primers for SrMV Screening

Based on the SrMV sequences (KJ541740.1, KM025053.1, KM025054.1, KM025050.1, NC_004035.1, and NP_734085.1), seven primer pairs were designed using Oligo 7 and Primer 5 software and synthesized by a commercial company. These primers were designed to target the conserved region of the P3 gene of SrMVs (Table 1). The seven primer pairs were then screened using the RAA Basic Nucleic Acid Amplification Kit to determine which primer has the best amplification effect. The amplification reactions were carried out following the instructions provided with the kit. The reaction conditions were set at 39 °C for 30 min using a constant-temperature metal bath (Shanghai Yiheng Technology Co., Ltd., Shanghai, China). Primer pairs with better amplification efficiency and specificity were used for further optimization of reaction conditions.

### 2.5. RT-RAA Assay and Reaction Conditions

An RT-RAA assay was performed using the RAA Basic Nucleic Acid Amplification Kit (Hangzhou Zhongce Biotechnology Co., Ltd., Hangzhou, China) to detect SrMV-positive cDNA. Add various ingredients into a small tube containing freeze-dried powder in the order recommended in the manual, including the A buffer (41.5 μL), 2 μL each of forward and reverse primers (10 μmol/μL), cDNA (2 μL), and the B buffer (2.5 μL, magnesium acetate solution). According to references and product instructions, the initial reaction was carried out at 39 °C for 30 min. After the process of amplification, the nucleic acid fragments were extracted using a DNA extraction solution (Beijing Suolaibao Company, Beijing, China) and detected on a 1.5% agarose gel.

Optimization experiments were conducted to determine the optimal RAA reaction conditions. Different temperature intervals (24 °C, 29 °C, 34 °C, 39 °C, 44 °C, and 49 °C) and different reaction times (5 min, 10 min, 20 min, 30 min, 40 min, 50 min, and 60 min) were tested. The dosage of magnesium acetate (280 mM/μL) was varied by 1.5 μL, 2 μL, 2.5 μL, 3 μL, and 3.5 μL. Various concentrations of forward and reverse primers (0.2 μmol/μL, 0.4 μmol/μL, 0.6 μmol/μL, and 0.8 μmol/μL) were tested.

### 2.6. Specificity of the RAA Method

The specificity of the established method was verified through cross-reactions using cDNA from various sources. These sources comprised samples of infected sugarcane plants with SrMV, the sugarcane mosaic virus (SCMV), sugarcane stripe mosaic virus (SCSMV)-infected samples, sugarcane yellow leaf virus (SCYLV), and virus-free sugarcane samples.

### 2.7. Sensitivity Comparison of RAA and RT-PCR

The rapid cloning reagent TA/Blunt-Zero Cloning Kit was used to construct the cloning vector for SrMV. The colonies containing the target sequence were screened and cultured, and plasmids were extracted using the RapidLyse Plasmid Mini Kit (Nanjing Novozyme Biotechnology Co., Ltd., Nanjing, China). The number of copies of the pUC57-P3 plasmid was determined, and then a 10-fold gradient with a concentration of 1 × 10^10^ copies/μL, 1 × 10^9^ copies/μL, 1 × 10^8^ copies/μL, 1 × 10^7^ copies/μL, 1 × 10^6^ copies/μL, 1 × 10^5^ copies/μL, 1 × 10^4^ copies/μL, 1 × 10^3^ copies/μL, 1 × 10^2^ copies/μL, and 1 × 10^1^ copies/μL was made. The positive standards with different concentrations were treated with RAA using the RAA basic kit. The sensitivity of the established approach was confirmed by comparing the RAA results with the results of the RT-PCR reaction.

### 2.8. Reliability of the RT-RAA Assay

To evaluate the reliability of this detection technique, a scientific method was followed. We randomly selected 90 sugarcane leaf samples from three fields, including samples with streaking mosaic symptoms and samples without symptoms. Both RT-RAA and RT-PCR techniques were employed simultaneously for SrMV detection. A probabilistic analysis was performed with a 95% confidence interval. Then, the κ and p values of the RT-RAA and RT-PCR were statistically calculated. The statistical analysis was performed using the statistical software package for the social sciences (SPSS) version 21.0 (IBM, Armonk, NY, USA).

## 3. Results

### 3.1. Optimization Results of RAA Reaction Conditions

**Figure 1:**
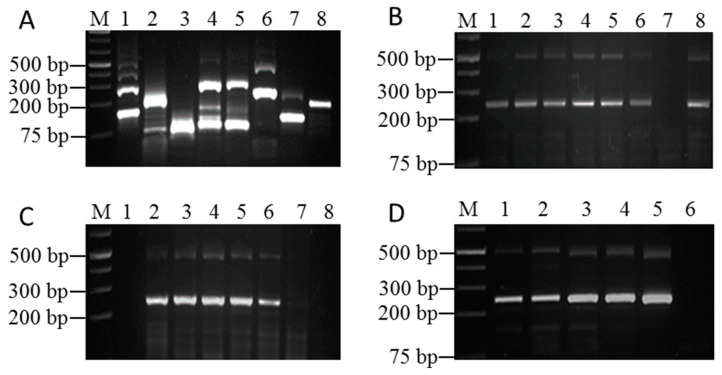
(**A**) Seven pairs of RAA primers for cDNA screening of SrMV-infected samples. M: 1 Kb plus Marker; 1: 2F/2R; 2: 3F/3R; 3: 4F/4R; 4: 5F/5R; 6F/6R; 6: 3PF/3PR; 7: 3P2F/3P2R; 8: positive reference (comes with the RAA kit and can determine the reliability of the reaction system). (**B**) Optimization of RAA reaction temperature. The reaction time is tentatively set at 30 min. M: 1 Kb Plus Marker: 1: 24 °C; 2: 29 °C; 3: 34 °C; 4: 39 °C; 5: 44 °C; 6: 49 °C; 7: negative control; 8: positive control. (**C**) Optimizing RAA reaction time. The reaction temperature was 39 °C. M: 1 Kb Plus Marker: 1: 5 min; 2: 10 min; 3: 20 min; 4: 3 0 min; 5: 40 min; 6: 50 min; 7: 60 min; 8: negative control. (**D**) Optimization of primer dosage. The reaction temperature is 39 degrees, and the reaction time is 30 min. M: 1 Kb Plus Marker: 1: 0.5 μL; 2: 1 μL; 3: 2 μL; 4: 3 μL; 5: 4 μL; 6: negative control.

According to the requirements of RNA primer design and the c-terminal conserved region of the virus P3 gene, seven pairs of candidate primers were designed (Table 1). After the initial primer screening on RT-PCR, we found that the amplification efficiency of primer pairs 4F/4R and 3PR/3PF was higher, and non-specific amplification was relatively low (Figure 1A). The two pairs of primers 4F/4R and 3PR/3PF were selected for further optimization experiments. However, primers 4F/4R formed more primer dimers at lower temperatures and mixed with target bands that were difficult to distinguish (data not shown). In the follow-up optimization experiment, the primer 3PR/3PF amplification fragment of the 244 bp band was obtained and confirmed after sequencing (Figure S1). We selected primers 3PR/3PF for this experiment. The maximum amplification efficiency was obtained at 39 °C in the optimization process of reaction temperature using a reaction time of 30 min (Figure 1B). In this study, RAA technology obtained effective results in 10 min (Figure 1C); this characteristic is superior to other detection technologies. High detection efficiency can be obtained within 20 to 40 min, of which 30 min may be the highest detection efficiency. Even for a lower viral load, the electrophoresis of the product showed the brightest band (Figure 1C). Furthermore, increasing the primer concentration improved the amplification efficiency; however, it also increased the heterogeneous bands (Figure 1D). Consequently, a final primer concentration of 0.4 μmol/μL, shown in Figure 1D, line 3, was determined for subsequent studies to obtain the best results.

### 3.2. Specificity of the RT-RAA Method

**Figure 2:**
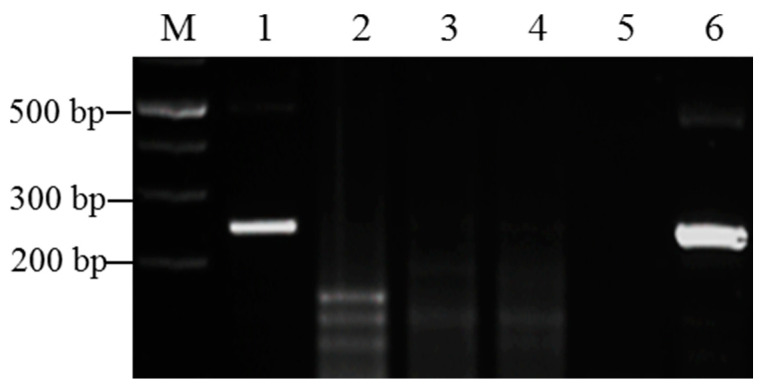
Evaluation of the specificity of the RAA method for SrMV detection. M: 1 Kb plus marker: 1: cDNA of a sugarcane sample infected with SrMV; 2: cDNA of a sugarcane sample infected with SCMV; 3: cDNA of a sugarcane sample infected with SCSMV; 4: cDNA of a sugarcane sample infected with SCYLV; 5: blank control (dd-water as the template); 6: plasmid-containing SrMV P3 gene as a positive control, reaction time 20 min, at 39 °C.

At present, the common sugarcane viruses in China include SrMV, SCMV, SCSMV, and the sugarcane yellow leaf virus (SCYLV). To test the specificity of the selected primers 3PR/3PF and the optimized reaction conditions, the RAA method was used to detect positive sugarcane leaves infected with SrMV, SCMV, SCSMV, and SCYLV, respectively. The results showed that the SCMV, SCSMV, SCYLV, and negative control did not appear in the target bands. Only the SrMV-positive samples showed target bands of approximately 244 bp (Figure 2). These results suggest that the existing RT-RAA detection method using the selected primers shows high specificity for SrMV.

### 3.3. Sensitivity Comparison of RT-RAA and RT-PCR

**Figure 3:**
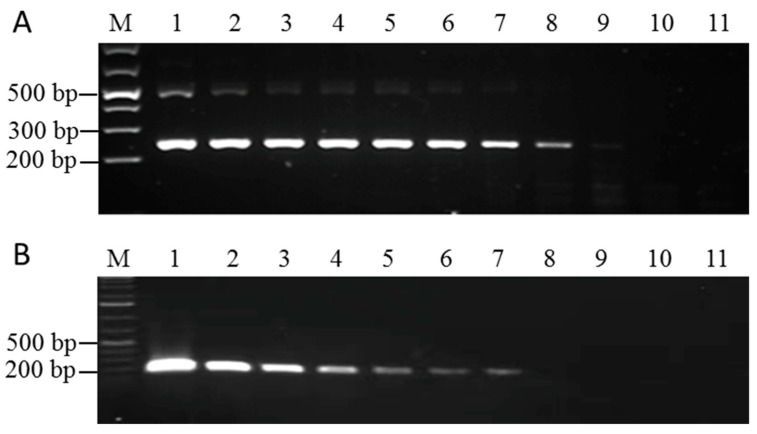
Sensitivity comparison between RAA and PCR for SRMV detection. (**A**) Determination of the sensitivity of the RAA using serially diluted standard plasmids. M: 1 Kb plus marker; line 1–10: 1 × 10^10^ copies/μL to 1 × 10^1^ copies/μL, respectively; 11: blank control (dd-water as the template). (**B**) Determination of the sensitivity of PCR reactions using serially diluted standard plasmids. M: 1 Kb plus marker; line 1–10: 1 × 10^10^ copies/μL to 1 × 10^1^ copies/μL, respectively; 11: blank control (dd-water as the template).

The sensitivity of the RAA method was assessed by comparing it with RT-PCR. The pUC57-P3 plasmid was serially diluted 10 times, and samples from each dilution were examined using both RT-PCR and RAA. The standard plasmid concentrations after dilution were 1 × 10^10^ copies/μL, 1 × 10^9^ copies/μL, 1 × 10^8^ copies/μL, 1 × 10^7^ copies/μL, 1 × 10^6^ copies/μL, 1 × 10^5^ copies/μL, 1 × 10^4^ copies/μL, 1 × 10^3^ copies/μL, 1 × 10^2^ copies/μL, and 1 × 10^1^ copies/μL. The results showed that SrMV could be detected at a concentration of 10^4^ copies/μL using RT-PCR and 10^2^ copies/μL on the RT-RAA. Therefore, it can be concluded that the RAA detection method was 100-fold more sensitive than PCR (Figure 3A,B).

### 3.4. Reliability of the RT-RAA Assay

To evaluate the reliability of RAA for detecting SrMV, a total of 90 field samples were analyzed, and the results were assessed in terms of percent agreement, sensitivity, specificity, and kappa (κ) value. The percent agreement between RT-RAA and RT-PCR was found to be 91.11% (Table 2), indicating a substantial level of concordance. The sensitivity of SrMV via RT-RAA was calculated to be 96.36% (95% CI: 86.39–99.37%), indicating its capability to accurately identify true-positive samples. Moreover, the specificity of the RT-RAA assay was found to be 82.86% (95% CI: 65.70–92.83%), highlighting its ability to correctly classify true-negative samples. The calculated κ value of 0.809 (*p* < 0.001) further confirmed the high level of agreement between the two methods. These findings demonstrate that RT-RAA is a reliable and robust diagnostic tool for the detection of SrMV. It offered rapid and accurate results, showing comparable performance to the well-established RT-PCR method. The use of RT-RAA for SrMV detection in sugarcane has the potential to dramatically improve disease surveillance and provide timely disease management techniques.

## 4. Discussion

SMD is among the most harmful and prevalent diseases in sugarcane planting regions all over the world and is a systemic disease that can be transmitted by infected seed canes. The prevalence of SMD can be caused by the long-term cultivation of limiting sugarcane cultivars and continuous cropping, as well as by insufficient quarantine efforts for the exchange of sugarcane planting materials across commercial sugarcane planting regions [3,19]. Symptoms of SMD can differ in intensity with sugarcane variety, growing conditions, and species, in addition to the strains of the causative virus [20]. Disease symptoms can be aggravated by the synergism of plant viruses. Single or mixed viral infections of sugarcane produce similar disease symptoms and are difficult to identify visually, which presents challenges for field diagnosis, prevention, and disease control [21]. The most cost-effective strategy is to breed cultivars resistant to SMD. Some varieties resistant to SrMV have been identified and evaluated for further use in breeding programs in the future [22]. The intensive planting of sugarcane has become popular due to the rapid growth of modern sugarcane planting industries [23]. However, most sugarcane planting areas generally reproduce through cane stems, which leads to the generational accumulation of viruses in cane stems. Therefore, it is particularly urgent to establish a method for the rapid identification and accurate diagnosis of SrMV.

In this research, we employed recombinase-mediated strand replacement nucleic acid amplification technology (RAA), which has primarily been used for the detection of animal pathogens [24,25]. The application of RAA to plant viruses is still in its infancy, and this technique has been utilized for the identification of SrMV. Compared to traditional PCR, RAA technology has many advantages in terms of overall reaction time and ease of experiment development. The primary RAA detection method established in this study uses recombinase, DNA polymerase, and single-strand binding proteins to facilitate DNA double-strand unzipping, extension, and amplification without the need for high-temperature denaturation. The equipment necessary for this process is minimal, requiring only a metal water bath, or an ordinary water bath, and a sufficient number of DNA fragments can be obtained in about 20 min. Subsequent identification can be performed using gel electrophoresis, real-time fluorescence, or a lateral flow dipstick.

In this study, compared with RT-PCR, the RT-RAA method showed some nonspecific amplification, and a band of about 500 bp appeared. Because of the negative control, this nonspecific amplification does not affect the judgment of positive results. We speculate that the nonspecific amplification by RAA was due to the use of relatively low amplification temperatures, such as 20~30 °C. However, the RT-PCR method uses an annealing temperature of 60 °C and an amplification temperature of 72 °C. This method has significant advantages as compared to traditional plant virus detection methods because the sensitivity of the RT-RAA detection method is significantly higher. Our sensitivity assay results showed that SrMV could be detected at a concentration of 10^4^ copies/μL using RT-PCR and 10^2^ copies/μL on the RT-RAA. The findings of this study show that RT-RAA is a strong and reliable diagnostic technique for SrMV detection. It provides accurate results, showing comparable performance to the well-established RT-PCR method.

The SrMV detection method based on RAA technology holds great potential and is suitable for rapidly screening a large number of samples. In this regard, the RT-RAA method is crucial for managing fields, controlling diseases, and implementing quarantine measures for the cultivation of healthy sugarcane and cane seeds.

## 5. Conclusions

This study established a rapid and effective isothermal nucleic acid amplification method (RAA) for detecting SrMV in sugarcane plants. Specific primers were designed and synthesized regarding the conserved part of the P3 gene of SrMV, serving as the target sequence. The RAA method was assessed for its temperature and time limits, specificity, and sensitivity. The results showed that this method exhibited flexibility regarding reaction temperature (24–49 °C) and effective amplification of the target sequence within 10 min at 39 °C. Moreover, this method demonstrated high specificity, as it did not show any cross-reactivity with other common sugarcane viruses with a detection limit of 10^2^ copies/μL standard plasmids. Thus, RAA is a reliable and efficient technique for detecting SrMV in sugarcane plants.

## Figures and Tables

**Table 1:** RAA primer sequence.

Primer	Primer Sequence	Product Length (bp)	Primer Length (bp)
2F	TATAAGCCACAACAGCAAGCATCTCCAAACA	142	31
2R	TGCACCATACCATTAGTCCGCTCATAACAAC		30
3F	TAGATGTTGATGTTGTAGTGGATTTCGGTC	189	30
3R	TCTCCTGTAGTCCTTTCATTGTCACACCCG		30
4F	ATAAGCCACAACAGCAAGACATTTCAAACA	140	30
4R	GCACCATACCATTAGTCCACTCATAACAACTG		32
5F	GCAAAGAGCACAAAATCAGAAAGATAAAGAC	296	31
5R	TTTCTATGCACCATACCATTAGTCCACTCAT		31
6F	ATGATGAAGCAGCAGAGAAACAGAGACAAG	298	30
6R	CGTGTATTTGAGATGTCTTGCTGTTGTGGC		30
3PF	AGTCAGCTCTATTTCAACCAAACTCCACCAC	244	31
3PR	TCTCACTTCGCTAACTTCTCGTTCGTATTCC		31
3P2F	GGAATACGAACGAGAAGTTAGCGAAGTGAGA	117	31
3P2R	CTTGGATGATTCTCTCTAATGTATGCTATG		30

**Table 2:** Comparison of two detection methods for field samples.

		RT-RAA		Total	Kappa (*κ*)	*p* Value of Kappa	Sensitivity% (95% CI)	Specificity% (95% CI)
		Positive	Negative					
RT-PCR	Positive	53	2	55	0.809	<0.001	96.36 (86.39–99.37)	82.86 (65.70–92.83)
	Negative	6	29	35				
Total		59	31	90				

## Data Availability

The data that support the findings of this study are available from the corresponding author upon reasonable request.

## Supplementary Materials

The following supporting information can be downloaded at: https://www.mdpi.com/article/10.3390/pathogens12081055/s1, Figure S1: The fragment amplified by RAA was recovered by gel electrophoresis, the recovered DNA fragment was cloned into T-vector and sequenced, the sequence (RAA-seq) obtained was aligned with two known SrMV isolates SrMV-xos and SrMV-yh.
