# Supplementary material for: Optimization and Validation of Reverse Transcription Recombinase-Aided Amplification (RT-RAA) for Sorghum Mosaic Virus Detection in Sugarcane
Source: Pathogens. 2023 Aug 18;12(8):1055. doi: 10.3390/pathogens12081055 (PMC10457762; doi:10.3390/pathogens12081055)
Supplement: Supplementary file 1 [file pathogens-12-01055-s001.zip › pathogens-2510589-supplementary.pdf]

Supplemental Figure S1

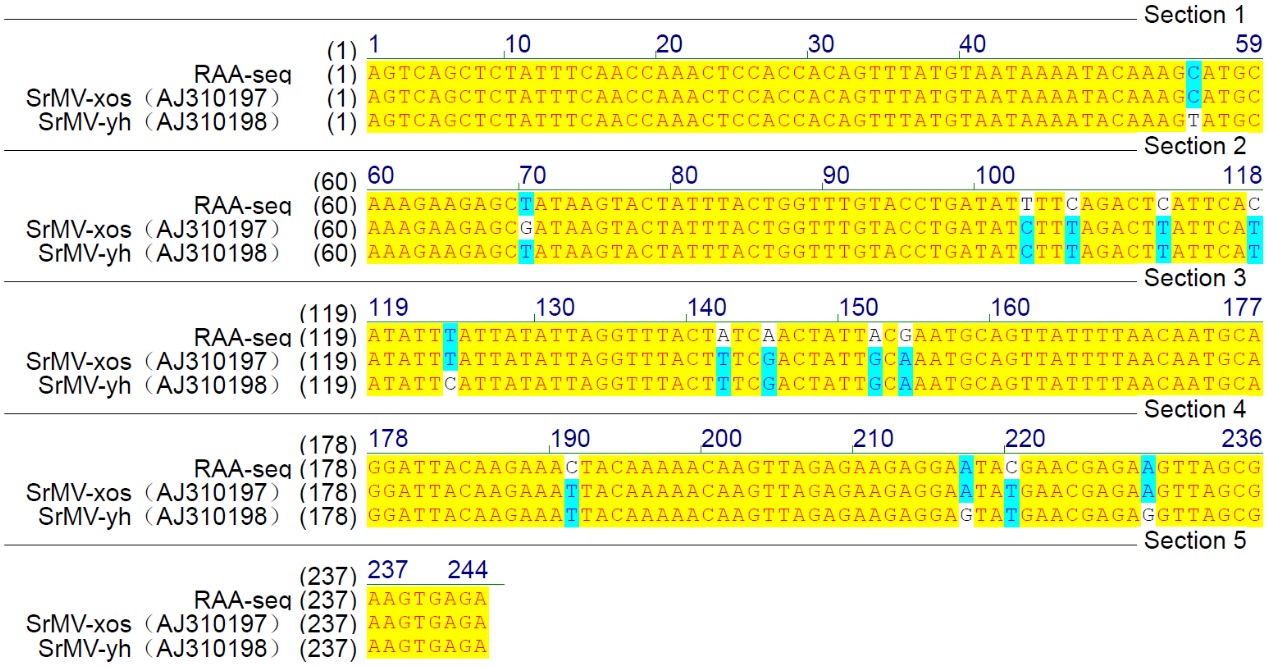

Figure S1. The fragment amplified by RAA was recovered by gel electrophoresis, the recovered DNA fragment was cloned into T-vector and sequenced, the sequence (RAA-seq) obtained was aligned with two known SrMV isolates SrMV-xos and SrMV-yh.
